# Molecular Profiling-Selected Therapy for Treatment of Advanced Pancreaticobiliary Cancer: A Retrospective Multicenter Study

**DOI:** 10.1155/2015/681653

**Published:** 2015-05-28

**Authors:** Ron Epelbaum, Einat Shacham-Shmueli, Baruch Klein, Abed Agbarya, Baruch Brenner, Ronen Brenner, Eliahu Gez, Talia Golan, Ayala Hubert, Ofer Purim, Mark Temper, Ella Tepper, Andreas Voss, Kenneth Russell, Addie Dvir, Lior Soussan-Gutman, Salomon M. Stemmer, Ravit Geva

**Affiliations:** ^1^Department of Oncology, Rambam Health Care Campus, 3109601 Haifa, Israel; ^2^Facutly of Medicine, Technion-Israel Institute of Technology, 3525406 Haifa, Israel; ^3^Division of Oncology, Sheba Medical Center Tel Hashomer, 5262100 Ramat-Gan, Israel; ^4^Sackler Medical School, Tel Aviv University, 6997801 Tel Aviv, Israel; ^5^Department of Oncology, Assuta Hospital, 6971028 Tel Aviv, Israel; ^6^Department of Oncology, Meir Medical Center, 4428164 Kfar Saba, Israel; ^7^Davidoff Center, Rabin Medical Center, 4941492 Petah Tikva, Israel; ^8^Department of Oncology, Wolfson Hospital, 5822012 Holon, Israel; ^9^Division of Oncology, Tel-Aviv Sourasky Medical Center, 6423906 Tel Aviv, Israel; ^10^Sharett Institute of Oncology, Hadassah Hebrew University Medical Center, 9124001 Jerusalem, Israel; ^11^The Hebrew University Hadassah Medical School, 9112102 Jerusalem, Israel; ^12^Caris Life Sciences, 4052 Basel, Switzerland; ^13^Oncotest-Teva Pharmaceutical Industries, Ltd., 60850 Shoham, Israel

## Abstract

This multicenter cohort study assessed the impact of molecular profiling (MP) on advanced pancreaticobiliary cancer (PBC). The study included 30 patients treated with MP-guided therapy after failing ≥1 therapy for advanced PBC. Treatment was considered as having benefit for the patient if the ratio between the longest progression-free survival (PFS) on MP-guided therapy and the PFS on the last therapy before MP was ≥1.3. The null hypothesis was that ≤15% of patients gain such benefit. Overall, ≥1 actionable (i.e., predictive of response to specific therapies) biomarker was identified/patient. Immunohistochemistry (the most commonly used method for guiding treatment decisions) identified 1–6 (median: 4) actionable biomarkers per patient. After MP, patients received 1–4 (median: 1) regimens/patient (most commonly, FOLFIRI/XELIRI). In a decision-impact analysis, of the 27 patients for whom treatment decisions before MP were available, 74.1% experienced a treatment decision change in the first line after MP. Twenty-four patients were evaluable for clinical outcome analysis; in 37.5%, the PFS ratio was ≥1.3. In one-sided exact binomial test versus the null hypothesis, *P* = 0.0015; therefore, the null hypothesis was rejected. In conclusion, our analysis demonstrated the feasibility, clinical decision impact, and potential clinical benefits of MP-guided therapy in advanced PBC.

## 1. Introduction

Pancreaticobiliary cancers are relatively rare malignancies. In the US, pancreatic cancer represents 3% of all new cancers and gallbladder/other biliary cancers represent 0.6% of all new cancers [1]. Despite its rarity, pancreatic cancer is responsible for 7% of cancer deaths [1], reflecting a need for better therapeutic approaches and better clinical outcomes in this disease. The 5-year survival rate for patients with early stage pancreatic cancer is less than 25%; once the disease metastasizes, it is uniformly fatal with a median overall survival (OS) of 6–11 months [2].

Gemcitabine-based treatment is the most common first-line therapy in locally advanced and metastatic pancreatic cancer [3]; however, most patients progress relatively quickly. In clinical trials and retrospective analyses of patients in clinical practice, 16–57% of gemcitabine-pretreated patients proceeded to receive second-line therapy [4–11]. Second-line regimens are potentially effective [12]; however, at present, treatment options are limited to a few drugs. The combination of fluorouracil (5-FU) and oxaliplatin has become a commonly used regimen in the second-line setting after a randomized trial in patients with gemcitabine-refractory pancreatic cancer demonstrated that the OFF/FF regimen (FF: 5-FU plus folinic acid or leucovorin (LV); OFF: FF plus oxaliplatin) was associated with a significantly longer progression-free survival (PFS) and OS compared with FF alone [13]. However, in a recent randomized phase 3 trial evaluating 5-FU/LV with or without oxaliplatin for the treatment of gemcitabine-refractory pancreatic cancer, adding oxaliplatin was not associated with PFS benefit [14]. Non-gemcitabine-based therapy such as folinic acid plus 5-FU plus irinotecan plus oxaliplatin (FOLFIRINOX) is an additional effective first-line treatment in the metastatic setting [15]. Data on second-line therapy after first-line treatment with FOLFIRINOX are limited [16, 17].

Precision treatment of cancer individualizes therapies according to the molecular profile of patients' tumors, as determined using methodologies such as immunohistochemistry (IHC), fluorescence/chromogenic* in situ* hybridization (FISH/CISH), microarray (MA) analyses, reverse transcription polymerase chain reaction (RT-PCR) analysis, and next-generation sequencing (NGS). This approach has made great progress in recent years due to advances in predictive biomarker research and the molecular understanding of cancer. Recently, molecular profiling- (MP-) guided treatment has proven to be an effective approach in advanced tumors [18–21]. Specifically, Von Hoff and colleagues, in their pilot study evaluating 66 patients with a variety of refractory cancers (including 2 with pancreatic carcinoma) whose treatment was MP-guided, demonstrated that this approach led to PFS that was ≥30% longer than the last regimen on which patients progressed (before MP) in 27% of patients [18].

The current study was designed to assess the MP-guided treatment approach using Caris Molecular Intelligence (CMI) tumor profiling service (Caris Life Sciences, Irving, TX) in a cohort of patients with advanced pancreaticobiliary cancer. Specifically, this study aimed to characterize the molecular profile of patients' tumors, to evaluate the impact of MP on clinical decision making, and to evaluate the potential clinical benefit of MP-guided therapy. Clinical benefit was assessed by comparing clinical outcomes using MP-guided therapy to those of the most recent regimen on which the patient experienced disease progression before MP.

## 2. Materials and Methods

### 2.1. Study Design and Patient Population

This was a multicenter retrospective study evaluating patients with advanced pancreaticobiliary cancer who (i) failed at least one line of therapy for their advanced disease before undergoing MP; (ii) had their tissue sample tested using CMI; and (iii) were treated with MP-guided therapy after MP. The study was approved by the institutional review boards of the participating institutions.

### 2.2. Data Source

Information on patients' baseline characteristics, physicians' initial treatment recommendations, actual treatments received, and clinical outcomes were collected from patients' files. Progression was determined based on clinical evaluation, imaging (mostly computed tomography (CT) and positron emission tomography (PET)/CT), and biomarker analyses (CA 19-9 and carcinoembryonic antigen (CEA)). CMI results were provided by Teva-Oncotest Pharmaceutical Industries Ltd., the representative of Caris Life Sciences in Israel.

### 2.3. Molecular Profiling

CMI analyses were performed in Caris Life Sciences laboratories (Phoenix, AZ) on paraffin-embedded tumor samples taken during (i) biopsies of the primary tumors, (ii) surgical procedures performed (e.g., Whipple procedure, total pancreatectomy), or (iii) biopsies of metastatic lesions.

The MP included IHC analysis of up to 18 biomarkers, FISH/CISH analysis of up to 3 biomarkers, gene expression MA analysis of up to 88 genes, RT-PCR analysis of 8 biomarkers, sequencing analysis by the Sanger method of up to 3 genes (epidermal growth factor receptor (EGFR), KRAS, and BRAF), and NGS of 45 genes. The types of analyses performed and the specific biomarkers tested depended on the amount of tissue sample available (i.e., if the amount was insufficient, the analyses were prioritized by the treating physician) and the specific timeframe in which the testing occurred. The panel of tests evolved over time as new biomarker information was published and taken into account by CMI. “Actionable” biomarkers were defined as those predictive of response to specific commercially available chemotherapeutics or biologic agents (the use of these agents in pancreaticobiliary cancer could be either on-label or off-label) [18].

### 2.4. Statistical Analysis

Descriptive statistics were used to summarize patient and tumor characteristics, planned treatment before MP, and actual treatments received. A treatment decision change was defined as any change from before MP recommendation to actual treatment received. These changes could include omitting/replacing/adding agents to a recommended regimen that was specified by the treating physician before MP, deciding on a specific regimen if before MP the treating physician was unsure which treatment should be administered or deciding to treat with an anticancer therapy if the treating physician initially recommended best supportive care.

MP-guided therapy was defined as having a clinical benefit if the PFS ratio between the longest PFS on MP-guided therapy and the PFS on the last therapy before MP was ≥1.3 (i.e., using patients as their own controls) [22, 23]. One-sided exact binomial test versus a null hypothesis of ≤15% of patients with PFS ratio ≥1.3 was performed at a significance level of 0.05 [18].

## 3. Results

### 3.1. Patient Disposition

**Figure 1:**
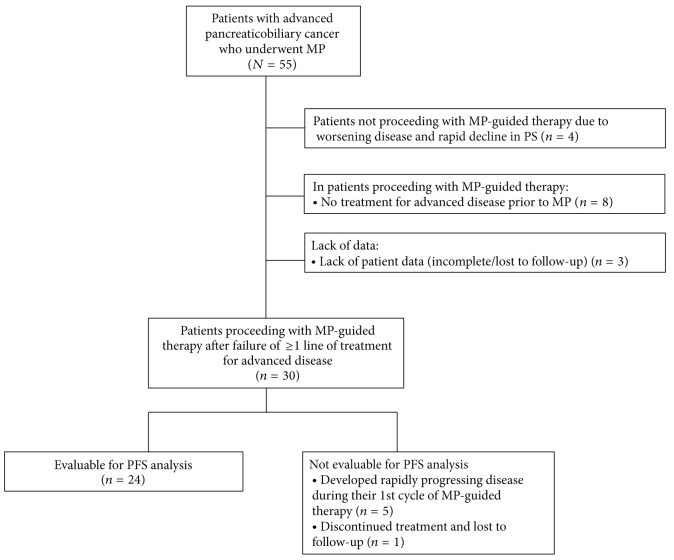
Patient disposition.

A total of 55 patients with advanced pancreaticobiliary cancer, who were treated in the participating institutions, underwent MP between July 2008 and February 2013. Of these 55 patients, 25 (45.5%) were excluded from the MP analysis, mostly because they did not proceed with MP-guided therapy due to worsening disease and rapid decline in PS or because they had no prior therapy for advanced disease (Figure 1). Thus, thirty patients (54.5%) were included in the MP analysis as they were treated with MP-guided therapy after failure of at least one line of treatment for their advanced disease. Six patients (10.9%) were further excluded from the PFS analysis, mainly because they developed rapidly progressing disease during their first cycle of MP-guided therapy, and therefore their PFS after MP could not be determined and compared with their last PFS before MP (Figure 1).

### 3.2. Baseline Patient and Tumor Characteristics

Baseline patient and tumor characteristics for the study group of 30 patients are presented in Table 1. Patients were mostly males (73.3%) and the median (range) age at diagnosis was 57 (29–80) years. Twenty-two patients (73.3%) had pancreatic cancer and 8 (26.7%) had biliary cancer. The majority of patients (60.0%) had Eastern Cooperative Oncology Group (ECOG) performance status (PS) value of 1. Before MP, patients received 1–4 treatment regimens for their advanced disease, with the majority of patients (63.3%) receiving 1 treatment regimen in this setting (6 patients progressed on adjuvant therapy, and their adjuvant gemcitabine monotherapy regimen was considered first-line treatment for advanced disease for the purpose of the current analysis). Together, the patients received 47 before-MP treatment regimens, including gemcitabine monotherapy (6), gemcitabine-based doublets (19), 5-FU/capecitabine-based doublets (10), FOLFIRINOX (3), 5-FU/capecitabine monotherapy, erlotinib monotherapy, poly(ADP-ribose) polymerase inhibitor (PARPi) monotherapy (2 regimens each), as well as 5-FU plus cisplatin plus epirubicin, and monotherapy regimens with docetaxel or cisplatin (1 regimen each).

### 3.3. MP Findings

In 15 patients (50%), MP was performed on samples derived from the primary tumor and in the remaining 15 patients (50%), MP was performed on samples derived from metastatic lesions (liver, 9 patients; soft tissue, 2 patients; appendix, small intestine, lymph node, and pancreas (in a biliary cancer patient), 1 patient each). Each patient's sample underwent 1–4 types of analyses (median, 2.5).

Physicians received reports specifying drug associations as known at the time of testing. Overall, at least one actionable biomarker was identified for each patient (median: 8.0; range: 1–22). In both IHC analyses (conducted for the entire cohort) and MA analyses (conducted for 17 patients), at least one actionable biomarker was identified for each patient (IHC: median, 4; range, 1–6; MA: median, 8; range, 3–20). Sequencing results were available for 13 patients (by the Sanger method for 12 patients and by NGS for 1 patient) and identified actionable biomarkers in 4 patients (30.8%). FISH/CISH results, which were available for 12 patients, did not identify any actionable biomarker. Sample from one patient underwent RT-PCR analysis which identified 3 actionable biomarkers.

The most common IHC-identified actionable biomarker (27 of 28 evaluated samples, 96.4%) was low/negative thymidylate synthase (TS), which may be associated with response to fluoropyrimidines and other folate analogs [24–26]. Other actionable biomarkers commonly identified by IHC included negative/low ribonucleotide reductase M1 subunit (RRM1; 23 of 26 evaluated samples, 88.5%), which may be associated with response to gemcitabine [27], and high topoisomerase 1 (TOPO1; 22 of 28 evaluated samples, 78.6%), which may be associated with response to irinotecan [28, 29] (Table 2).

The most common MA-identified actionable biomarker (13 of 17 evaluable patients, 76.5%) was overexpression of the gene for topoisomerase II alpha (*TOP2A*), which may be associated with response to anthracyclines [30, 31]. Other common actionable biomarkers included overexpression of the hypoxia-inducible factor 1-alpha gene (*H1F1A*; 9 of 17 evaluable patients, 52.9%), which may be associated with response to sorafenib [32], and overexpression of the gene for beta-type platelet-derived growth factor receptor (*PDGFRB*; 9 of 17 evaluable patients, 52.9%), which may be associated with response to imatinib [33] (Table 2).

Of the 13 patients for whom* KRAS* sequencing was performed, 4 patients (30.8%) had wild-type* KRAS*, which is associated with response to anti-EGFR therapy in colorectal cancer [34, 35]. In the sample that underwent RT-PCR analysis, the 3 actionable biomarkers included low TS, low RRM1, and high TOP2A.

### 3.4. Treatments Received after MP: The Impact of MP on Clinical Decision Making

In total, after MP, 47 treatment regimens were administered to the 30 evaluated patients, with a median (range) of 1 (1–4) line of therapy per patient. The median (range) duration between collecting the sample used for MP and the initiation of MP-guided therapy was 9.5 (0.8–45.2) months. The treatments administered (Table 3) were mostly selected based on IHC findings (MA findings, sequencing results, and RT-PCR findings impacted treatment selection in one patient each). Treatments included both drugs and regimens that are commonly used in this setting (e.g., 5-FU plus irinotecan (FOLFIRI), capecitabine plus irinotecan (XELIRI), and gemcitabine plus oxaliplatin (GEMOX)) and drugs that are not used in clinical practice in this setting such as sorafenib which is Food and Drug Administration- (FDA-) approved for renal cell carcinoma and hepatocellular carcinoma [36] and temozolomide which is FDA-approved for glioblastoma multiforme and anaplastic astrocytoma [37].

Information on treatment recommendations prior to the MP report was available for 27 patients. In 20 of these patients (74.1%), a treatment recommendation change was noted in the first after-MP treatment. These 20 patients included 12 patients where the treatment recommendation change included omitting/replacing/adding agents to the specific regimen that was recommended by the treating physician before MP; 2 patients where the treating physician was unsure which treatment to administer and the change entailed a decision on a specific regimen; and 6 patients, where the treating physician recommended best supportive care, and the change included a treatment with an anticancer therapy.

### 3.5. MP and Clinical Outcomes

**Figure 2:**
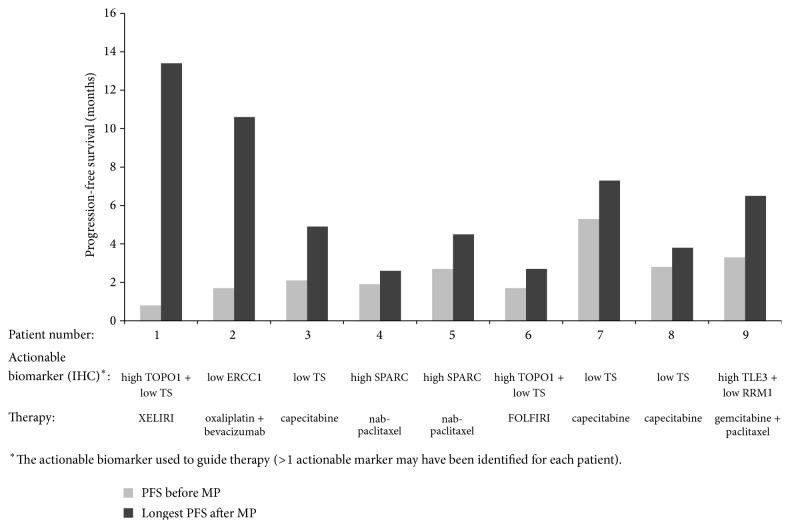
Comparison between the longest PFS on MP-guided therapy (dark grey) and PFS on last regimen on which patients progressed before MP (light grey) in 9 patients for whom this ratio was ≥1.3.

Twenty-four patients were included in the PFS analysis. The median (range) PFS on their last before MP treatment was 3.3 (0.8–23.1) months. In the first after MP treatment (24 evaluable patients), the median (range) PFS was 2.4 (0.8–10.6) months, and in the second treatment (8 evaluable patients) it was 2.1 (1.6–13.4) months. Together, the 24 patients had 40 lines of treatment after MP, with a median (range) PFS of 2.0 (0.8–13.4) months. In 9 of the 24 evaluable patients (37.5%), the ratio between the longest PFS on MP-guided therapy and the PFS on their last before MP regimen was ≥1.3 (Figure 2). These 9 patients had a median (range) PFS of 2.1 (0.8–5.3) months in their last regimen before MP and 4.9 (2.6–13.4) months in their longest PFS after MP. They received (after MP) various drugs/regimens including capecitabine monotherapy (3 cases), FOLFIRI/XELIRI (2 cases), nab-paclitaxel monotherapy (2 cases), gemcitabine with paclitaxel, and oxaliplatin with bevacizumab (1 case each) (Figure 2). A one-sided exact binomial test performed versus a null hypothesis of 15% or less of patients having PFS ratio ≥1.3 reached statistical significance (*P* = 0.0015) and the null hypothesis was rejected. Patients with PFS ratio of <1.3 and ≥1.3 received a similar number of treatments for advanced disease before MP (mean (SD) of 1.5 (0.8) and 1.6 (0.7), resp.).

## 4. Discussion

This retrospective study demonstrated the feasibility of the MP-guided therapy approach for patients with advanced pancreaticobiliary cancer. For each patient, at least one potentially actionable biomarker was identified, with IHC identifying 1–6 actionable biomarkers per patient (most commonly, negative/low TS, negative/low RRM1, and high TOPO1), all of which were observed in more than half of evaluable patients. Identifying actionable biomarkers impacted treatment decisions in the majority of patients (74%) and the modified treatment regimens were associated with clinical benefit in 37.5% of patients (statistically significantly more than 15%).

IHC was the most common methodology used for MP in our study. Other technologies like NGS which has become increasingly used in translational oncology may not be therapeutically relevant in pancreatic cancer. In this disease, KRAS mutations, which currently are not successfully targeted, are very common, occurring in the majority of patients (reviewed by Chiu et al. [38]). In a recent analysis of 2,400 pancreatic cancer patients of whom 82% were found to have mutated KRAS, mutations in BRAF, EGFR, HER2, FLT3, HRAS, PDGFRA, and PTEN were identified exclusively in KRAS wild-type patients, and even there, only rarely (8%) [39]. Thus, in pancreatic cancer, sequencing is unlikely to identify actionable mutations and IHC remains the methodology of choice for MP.

MP led to administration of commonly used drugs/regimens in the advanced pancreaticobiliary setting (e.g., FOLFIRI, XELIRI, GEMOX) as well as drugs that are not used in clinical practice in this setting (e.g., sorafenib and temozolomide). Although the use of the latter drugs was not associated with favorable clinical outcomes, the small number of patients treated with these drugs limits our ability to draw specific conclusions.

Overall, the findings of our study are consistent with those of recent studies describing the potential benefit of MP-guided therapy including a pilot trial conducted by Von Hoff and colleagues in 66 patients with a variety of refractory solid tumors and recent studies in patients with previously treated metastatic pancreatic cancer, metastatic breast cancer, or rare/advanced refractory cancers [18–21]. Notably, in the pancreatic cancer study involving 49 heavily pretreated metastatic patients, IHC identified at least 2 actionable biomarkers in most patients, and clinical activity was demonstrated (median OS of 5 months) [20]. Our findings are also consistent with recent studies describing the molecular makeup of pancreatic tumors from 1,029 patients and 2,400 patients [39, 40].

Advanced pancreaticobiliary cancer may be a good model to demonstrate the utility of the MP-guided approach in the second-line setting: almost all patients fail first-line systemic therapy relatively quickly; nonetheless, up to 57% of patients are willing and fit enough to pursue second-line treatment at disease progression [4–11]. However, evidence for the efficacy of various regimens in this setting is limited and no standard of care currently exists [41]. Studies suggest that patients gain clinical benefit from second-line chemotherapy and a recent retrospective analysis of 10 prospective randomized controlled trials demonstrated that second-line treatment was an independent predictor of OS and that approximately half of the median OS of patients with advanced pancreatic cancer was attributed to second-line therapy [42]. Furthermore, in recent years, the unmet clinical need in advanced pancreaticobiliary cancer has intensified, as nowadays more advanced patients have good PS and may benefit from second-line therapy. That is because of increasingly effective first-line treatment options and advances in imaging and relevant biomarker assessment that leads to earlier detection of progression. These characteristics of the advanced pancreaticobiliary cancer patient population along with the availability of multiple targets and potential regimens make MP-guided therapy a promising approach that could potentially optimize patient care.

Although promising, the MP-guided therapy approach has a number of limitations. For example, there may be additional biomarkers that were not tested and may be more appropriate for the individual patient. In addition, intrapatient/intratumor heterogeneity may lead to discordant responses and redundancy in signal-transduction pathways. Only for IHC and FISH/CISH it is easy to differentiate between the tumor cells and adjacent cells (e.g., tumor-infiltrating immune cells); for gene expression or sequencing analyses, a microdissection needs to be performed under supervision of skilled pathologists. Furthermore, pharmacokinetic effects on drug distribution as well as the effect of the tumor microenvironment are not taken into account by MP. All of these factors could influence the effectiveness of the chosen therapy.

The current study has a number of limitations. It is a retrospective, nonrandomized study, with a limited sample size, and the use of PFS ratio as an endpoint is relatively new and is a subject of debate [18, 22, 23, 43]. Furthermore, PFS determination could have been biased as patient monitoring was not standardized. Our study was also limited by the types of analyses performed and the heterogeneity in the MP analyses that stemmed from limitations associated with the amount of tissue available for each patient (i.e., if the amount was insufficient, only a subset of analyses was performed) as well as changes in the panel of tests performed over time due to advances in technology and accumulating evidence linking biomarkers and response/resistance to treatment. Notably, the MP analyses performed in our study did not include (for the most part) sequencing and therefore, mutations (either somatic or germline mutations with relevant familial implications) were not identified. Another source of heterogeneity in our study is the source of the tissue sample used for MP (primary tumors versus metastases) and the duration of time between collecting the sample for MP and the initiation of the MP-guided treatment. In several cases, this duration was long because a new biopsy was not performed prior to MP, most commonly due to the lack of tissue accessibility and poor PS of the patient. As tumors are known to evolve over time [44], use of the primary tumors for guiding treatment of a metastatic disease and a longer duration between tissue sampling and treatment initiation may reduce the effectiveness of the MP-guided therapy approach. In our study, there was no imbalance between primary and metastatic source of tissue for MP between the 9 patients with clinical benefit and the other patients. An additional limitation is the potential for selection bias, as CMI is not covered under the Israeli National Health Insurance Law and therefore patients may have elected not to undergo testing for financial reasons. The strengths of this study include its well-defined cohort of patients with pancreaticobiliary cancer and its representation of real-life clinical practice.

Given that, for patients with advanced disease who failed ≥1 line of therapy, no standard of care exists, the treating oncologist has a few alternatives. Either a treatment can be selected based on chance or prior personal experience in other patients or MP can be performed to generate a view of the molecular properties of a particular patient's tumor. In this context, even biomarkers that have only a low level of evidence linking them with specific therapeutics or evidence that was generated in another type of cancer become highly relevant for treatment selection. We suggest that this study is hypothesis-generating with respect to the role of MP-directed treatment decisions in pancreaticobiliary cancer. The strength of this approach depends on the evolution and validation of predictive markers. Notably, our findings suggest that MP-guided therapy may be more beneficial in earlier lines of advanced disease, as many patients in our study progressed rapidly and were therefore unable to receive such therapy.

## 5. Conclusions

Our retrospective analysis of a well-defined cohort of 30 patients with advanced pancreaticobiliary cancer demonstrated the feasibility of the MP-guided therapy approach in clinical practice and showed that MP influenced treatment decisions in the majority of patients. Over one-third of the patients experienced clinical benefit under MP-guided therapy (PFS ratio of ≥1.3), suggesting that this approach might be clinically beneficial. Further studies are warranted to explore the predictive power of MP in pancreaticobiliary cancer.

## Figures and Tables

**Table 1:** Baseline patient and tumor characteristics.

Characteristic	*N* = 30
Gender, *N* (%)	
Male	22 (73.3)
Female	8 (26.7)
Age,^1^ years	
Median (range)	57 (29–80)
Tumor type, *N* (%)	
Pancreatic cancer	22 (73.3)
Biliary cancer	8 (26.7)
Performance status (ECOG),^2^ *N* (%)	
0	1 (3.3)
1	18 (60.0)
2	10 (33.3)
Unknown	1 (3.3)
Number of lines of therapy for advanced disease before MP,^3^ *N* (%)	
1	19 (63.3)
2	7 (23.3)
3	2 (6.7)
4	2 (6.7)

^1^At diagnosis.

^2^At MP.

^3^For 6 patients who progressed on adjuvant therapy, their adjuvant regimen was considered first-line treatment for advanced disease.

ECOG: Eastern Cooperative Oncology Group; MP: molecular profiling.

**Table 2:** Actionable biomarkers (i.e., biomarkers predictive of response to specific therapies) identified by immunohistochemistry and microarray analysis.

Target	Number of patients out of evaluable patients (*N*/*N*)	Frequency, %
Immunohistochemistry		
Negative/low TS	27/28	96.4
Negative/low RRM1	23/26	88.5
High TOPO1	22/28	78.6
Negative/low ERCC1	19/26	73.1
High EGFR	3/5	60.0
Positive TLE3	3/6	50.0
High SPARC^1^	12/30	40.0
Negative/low MGMT	11/29	37.9
High PDGFR	5/17	29.4
High TOPO2A	5/25	20.0
High c-Kit	4/24	16.7
Positive PgR	2/27	7.4
Positive HER2	0/30	0.0
Positive ER	0/27	0.0
Positive AR	0/27	0.0
Microarray analysis		
*TOP2A* overexpression	13/17	76.5
*HIF1A* overexpression	9/17	52.9
*PDGFRB* overexpression	9/17	52.9
*SRC* overexpression	8/17	47.1
*TOP2B* overexpression	7/17	41.2
*VDR* overexpression	7/17	41.2
*RRM2B* underexpression	6/17	35.3
*ASNS* underexpression	5/17	29.4
*BRCA1* underexpression	5/17	29.4
*BRCA2* underexpression	5/17	29.4
*KIT* overexpression	5/17	29.4
*PDGFRA* overexpression	5/17	29.4
*ERCC1* underexpression	4/17	23.5
*MGMT* underexpression	3/17	17.6

^1^SPARC levels were considered high if either of the analyses (using monoclonal or polyclonal antibodies) demonstrated high SPARC expression levels.

5-FU: 5- fluorouracil; AR: androgen receptor; ASNS: asparagine synthetase; BRCA 1/2: breast cancer 1/2, early onset; EGFR: epidermal growth factor receptor; ER: estrogen receptor; ERCC1: excision repair cross complementation 1; FISH: fluorescent *in situ* hybridization; HER2: human epidermal growth factor receptor 2; HIF1A: hypoxia-inducible factor 1-alpha; IHC: immunohistochemistry; MGMT: O-6-methylguanine-DNA methyltransferase; PDGFR: platelet-derived growth factor receptor; PgR: progesterone receptor; RRM1: ribonucleotide reductase M1 subunit; RRM2B: ribonucleotide reductase M2 B; SPARC: secreted protein acidic, rich in cysteine; TLE3: transducin-like enhancer of split 3; TOP2B: topoisomerase II beta; TOPO1: topoisomerase 1; TOPO2A: topoisomerase IIA; TS: thymidylate synthase; VDR: vitamin D receptor.

**Table 3:** Chemotherapy regimens received after molecular profiling.

Treatment	Number	Frequency %
*Combination therapy *		
FOLFIRI/XELIRI	16	34.0
GEMOX	3	6.4
FOLFOX/XELOX	2	4.3
FOLFIRI + cetuximab	2	4.3
Capecitabine + cisplatin	1	2.1
5-FU + mitomycin	1	2.1
5-FU + adriamycin + methotrexate	1	2.1
Gemcitabine + nab-paclitaxel	1	2.1
Gemcitabine + paclitaxel	1	2.1
Oxaliplatin + bevacizumab	1	2.1
Pegylated liposomal doxorubicin + cetuximab	1	2.1

*Monotherapy *		
5-FU/capecitabine	5	10.6
Nab-paclitaxel	5	10.6
Sunitinib	1	2.1
Cetuximab	1	2.1
Gemcitabine	1	2.1
Sorafenib	1	2.1
Temozolomide	1	2.1
Mitomycin	1	2.1
Everolimus	1	2.1

**Total of regimens received **	**47**	**100**

5-FU: 5-fluorouracil; FOLFIRI: 5-fluorouracil/irinotecan; FOLFOX: 5-fluorouracil/oxaliplatin; GEMOX: gemcitabine/oxaliplatin; XELIRI: capecitabine/irinotecan; XELOX: capecitabine/oxaliplatin.
